# Raised CA19–9 and CEA have prognostic relevance in gallbladder carcinoma

**DOI:** 10.1186/s12885-020-07334-x

**Published:** 2020-08-31

**Authors:** Ashish Sachan, Sundeep Singh Saluja, Phani Kumar Nekarakanti, Nimisha, Bhawna Mahajan, Hirdaya H. Nag, Pramod K. Mishra

**Affiliations:** 1 Department of Gastrointestinal Surgery, Govind Ballabh Pant Institute of Post Graduate Medical Education and Research, Room no 218, Jawahar Lal Nehru Marg, New Delhi, 110002 India; 2 Department of Biochemistry, Govind Ballabh Pant Institute of Post Graduate Medical Education and Research, New Delhi, India

**Keywords:** Gall bladder cancer, CEA, CA19–9, Biliary cancer, Radical cholecystectomy

## Abstract

**Background:**

Role of tumor markers in gall bladder carcinoma (GBC) is not well established. We evaluated the prognostic value of carbohydrate antigen 19–9 (CA19–9) and carcinoma embryonic antigen (CEA) in patients with GBC.

**Methods:**

Of the 225 patients of GBC enrolled,176 patients were included in the study (excluded 49 patients with jaundice). Patients were divided into 3 groups; resectable *n* = 92, unresectable *n* = 17, metastatic *n* = 67. The clinico-pathological characteristics, tumor markers and survival data were analysed. The cutoff values of CA19–9 & CEA for predicting metastases were computed using receiver operating characteristic curve. Kaplan Meir survival and Cox regression analysis were done for factors predicting survival and recurrence.

**Results:**

The median value of Ca19–9 was significantly higher in metastatic group [resectable: 21.3, unresectable: 53.9 and metastatic: 79; *p* < 0.001] but not for CEA [3.5, 7.8 and 5 ng/ml (*p* = 0.20)]. A cutoff value of 72 IU/ml for CA19–9, 5 ng/ml for CEA had a sensitivity and specificity of 52 and 80%, 51 and 72% respectively for detection of metastatic disease. Median, 3-year & 5-year survival were significantly lower in patients with CEA > 4 (*p* = 0.041), Ca19.9 > 37 (*p* = 0.019), T3/T4 (*p* = 0.001), node positive (*p* = 0.001) and presence of perineural invasion (*p* = 0.001). However, on multivariate analysis, only Ca19.9 > 37 predicted recurrence (*p* = 0.002, HR 5.8).

**Conclusions:**

Raised CA19.9 and CEA predict metastatic disease in patients with GBC without jaundice with a high specificity and may help in prognostication of the patient. CA19–9 was better than CEA in prediction of tumor burden and in predicting recurrence.

## Background

Gall bladder cancer (GBC) is the most common malignancy of the biliary tract and constitutes 80–95% of the biliary tract cancers [1]. It is an aggressive tumor diagnosed at an advanced stage in more than 50% cases resulting in a poor outcome [2]. Although improvements in the imaging techniques have helped in diagnosing and staging in GBC, 20–40% patients of GBC with metastasis are missed [3, 4]. As per NCCN guidelines, CEA and CA19–9 are baseline tests and should not be done to confirm diagnosis [5]. Role of CEA and CA19–9 in detection of locally advanced or metastatic disease and prognosis of the disease is not well studied. Few studies have previously reported the prognostic and/or diagnostic value of CEA or CA19–9 [6–8] and the combined value of these tumor markers [9, 10]. However, no strong recommendation is available regarding their use in GBC. We conducted a study to determine the value of CEA and CA19–9 in detecting the advanced (locally advanced or metastatic) GBC and to identify their prognostic value in patients of GBC without jaundice.

## Methods

### Patients and methods

Patients with suspected GBC managed from October 2013 to April 2017 were reviewed from prospectively maintained database.

Informed patient consent was obtained for this research.

#### Inclusion criteria

Patients with GBC without surgical obstructive jaundice (SOJ) were included.

Patients with GBC with SOJ and GBC masquerades who underwent surgical resection for suspected GBC but had benign disease on final histopathology were excluded.

Patients were divided into three groups- resectable, unresectable (because of locally advanced disease) and metastatic disease.

### Definitions

#### Resectable disease

Patients with GBC without any evidence of unresectability or metastasis underwent curative resection.

#### Unresectable disease

Presence of main portal venous involvement or contralateral hepatic artery involvement either on dual phase contrast CT scan or as observed at operation.

#### Metastatic disease

Presence of biopsy proven inter-aortocaval (IAC) or lymph nodes on left side of coeliac axis, liver metastasis, omental, ovarian or peritoneal deposits were considered as metastatic disease.

##### Preoperative work up

All patients had a detailed clinical evaluation, blood investigations including tumor markers-CEA and CA19–9 and imaging. Chest X-ray, ultrasonography of abdomen with Doppler, dual phase contrast enhanced CT scan (CECT) were routinely done. Upper gastrointestinal endoscopy was done selectively in patients suspected to have gastro-duodenal infiltration. The extent of the disease and Fine needle sampling of Interaortocaval region was done with endoscopic ultrasound. PET CT scan was done in selected patients. AJCC 8th edition was used for staging [11].

#### Tumor markers

Serum CEA and CA19–9 levels were detected by electro-chemiluminescence immunoassay (Cobas; Roche Diagnostics, Germany) at the Department of biochemistry, GIPMER. Normal reference value for CEA and CA19–9 were (0-4 ng/ml) and (0–37 U/ml) respectively.

##### Peri-operative strategy

Patients with resectable disease at outpatient evaluation were admitted for definitive treatment. All patients underwent staging laparoscopy to rule out metastatic disease. Definitive procedure was abandoned if metastases was confirmed on biopsy. Frozen histopathological examination of Inter aortocaval lymphnode sample was performed prior to assessment of local resectability. Patients with unresectable disease underwent palliative bypass if required. To obtain R0 status, gallbladder mass with 2 cm liver wedge or segment IVB, V was resected. Patients with T4 disease underwent additional segmental/ sleeve resection of bowel or segmental resection of bile duct to achieve R0 status. Lymph nodes along the hepatoduodenal ligament, pericholedochal, periportal, peripancreatic and right celiac nodes were cleared as standard lymphadenectomy. Patients with infiltration of the pedicle on the right side underwent extended right hepatectomy. Frozen examination of cystic duct margin was a routine practice. Common bile duct resection was done in patients with obvious infiltration of duct by tumor, presence of malignancy at cystic duct margin on frozen examination and for lymph nodal clearance, if required. .

### Follow up

Patients were assessed clinically; biochemical including liver function tests and ultrasonography of the abdomen at 3 months interval during first year and 6 months interval in later years.. A CECT scan of the abdomen was done at 1 year in patients who remained well. It was done earlier if necessitated by clinical or ultrasound findings.

### Chemotherapy

Patients were treated by multimodality approach with gemcitabine-based chemotherapy in both the adjuvant and palliative setting. Any node positive tumor or T1b disease and above patients were subjected to adjuvant chemotherapy. In Palliative setting maximum possible supportive care was given. .

### Parameters recorded

Clinical and pathological details were collected, including age, gender, serum CEA and CA19–9 levels, Resectable or unresectable or metastatic disease status, resection margin status, AJCC staging of the disease, location of metastasis, survival time, survival status, adjuvant or palliative chemotherapy.

### Statistical analysis

Overall survival was measured from the time of resection to death or last followup. All data are presented as median for non-parametric distributions. Statistical analysis was done using SPSS software (IBM SPSS Statistics for Windows, Version 22.0. Armonk, NY: IBM Corp). Categorical variables were compared with the χ2 test or Fisher’s exact test. The cut off value of CA19–9 and CEA were calculated using receiver operating characteristic (ROC) curve in predicting metastatic disease. ROC curve was used to calculate the sensitivity and specificity of the tumor markers. The area under the ROC curve (AUROC) was adopted for the prognostic accuracy. A *P* value less than 0.05 was considered to indicate statistical significance. Survival analysis was done using Kaplan Meier test and comparison of survival between two groups was done using log rank test. Calculation of hazard ratio, univariate and multivariate analysis was done using Cox regression model.

## Results

A total of 225 patients with GBC were evaluated from October 2013 to April 2017. Forty-nine patients who presented with jaundice were excluded. Among the 176 patients included, 92 (52.3%) patients had a resectable disease, 17 (9.6%) were locally advanced & unresectable and 67 (38.1%) had metastatic disease. Among 84 patients with inoperable disease, 45 (14 locally advanced & unresectable; 31metastatic) were diagnosed preoperatively while 39 (3 unresectable; 36 metastatic) were diagnosed intra-operatively. Clinical and pathological variables of the study group are shown in Table 1.

**Table 1:** Shows clinicopathological characteristics of GBC patients

Variables	*n* = 176
Age in years median (Range)	55 (17–80)
Gender, n (%)	Male: 38 (21.6%) Female: 138 (78.4%)
Serum CEA, ng/ml median (Range)	4.2 (0.5–304.2)
Serum CA19–9, IU/ml median (Range)	28.2 (0.6–38,689)
Disease status, n (%)	Resectable: 92 (52.3%)
	Unresectable: 17 (9.6%)
	Metastatic: 67 (38%)
AJCC staging, n (%)	Stage 1: 16 (9.1%)
	Stage 2: 25 (14.2%)
	Stage 3: 43 (24.4%)
	Stage 4A: 17 (9.7%)
	Stage 4B: 75 (42..6%)

**Fig. 1:**
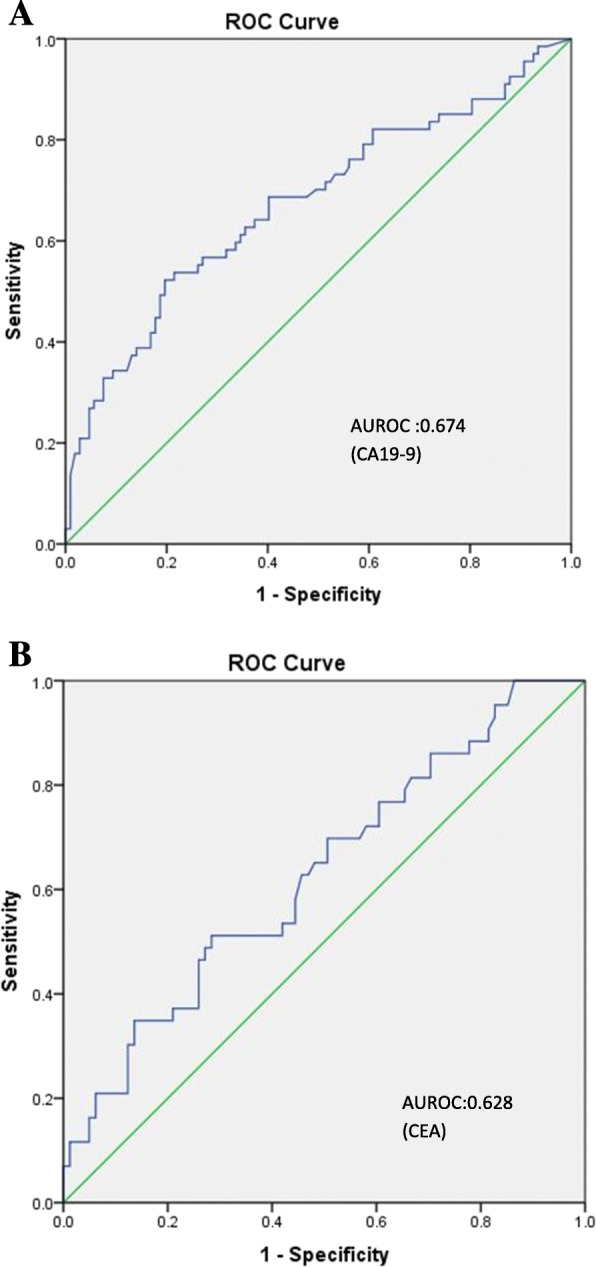
**a**: ROC curve for CA19–9 for detection of metastasis (Area under curve: 0.674 with a cut-off value of 72 IU/ml). **b**: ROC curve for CEA for detection of metastasis (Area under curve: 0.628 with a cut-off value of 5 ng/ml)

The median value for CA19–9 was significantly higher for patients with metastatic disease as compared to unresectable or resectable disease (79 vs 53.9 vs 21.35 IU/ml; *p* < 0.001). The median value for CEA was not statistically higher for patients with metastatic disease as compared to unresectable or resectable disease (5.1vs 7.85 vs 3.52 ng/ml; *p* = 0.20).(Table 2) A cut off value of 72 IU/ml for CA19–9 had a sensitivity of 52% and a specificity of 80% with area under ROC curve (AUC) 0.674 in predicting the metastatic disease, whereas a cutoff value of 5 ng/ml for CEA had a sensitivity of 51% and a specificity of 72% with AUC 0.628 for detection of metastatic disease. (Table 3, Fig. 1a and b). Only 14/56 patients (25%) with Ca 19.9 levels > 72 ng/ml whereas 14/45 patients (37%) with CEA levels > 5 ng/ml were resectable.

**Table 2:** Ca19.9 and CEA values among the three groups of patients

Variables	Group1(Resectable)	Group2(Unresectable)	Group3(Metastatic)	***P*** value
Ca19.9 median IU/ml	21.35	53.9	79	< 0.001
CEA median IU/ml	3.5	7.8	5.1	0.201

**Table 3:** Cutoff value with sensitivity and specificity of tumor markers for detection of metastasis

Tumor marker	Cut off value	Sensitivity	Specificity
**CA19–9**	72 IU/ml	52%	80%
**CEA**	5 ng/ml	51%	72%

**Fig. 2:**
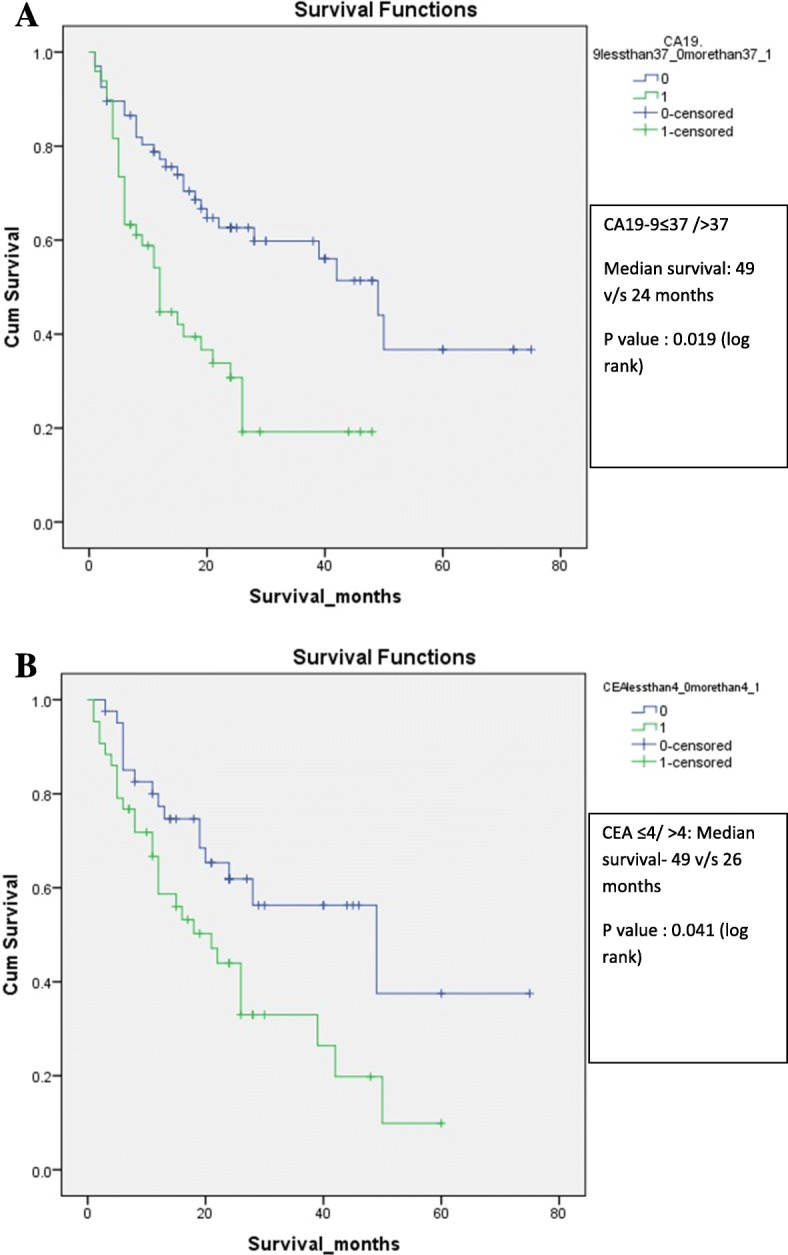
**a**: Kaplan Meir Survival graph showing significantly better survival for patients CA 19–9 ≤ 37 IU/ml as compared to CA 19–9 > 37 IU/ml. **b**: Kaplan Meir survival graph showing significantly better survival for patients CEA ≤4 ng/ml as compared to CEA > 4 ng/ml

Out of 176 patients, 92 patients had resectable disease and underwent curative resection. Except one patient, all had R0 resection. One patient developed surgical complication in the postoperative period and died during the hospital stay and was excluded for the survival analysis. Five patients were lost to follow up. Among remaining 86 patients, forty six patients received chemotherapy while 40 patients did not receive chemotherapy. The median survival of patients who received chemotherapy was similar those who did not receive chemotherapy group (30 months (95% CI:24–72) vs 29 months (95% CI: 24–75) *p* value = 0.669). The chemotherapy did not show significant difference in survival in the resectable group. Factors affecting the overall survival in the resectable group of patients (*n* = 86) were analysed. Median survival of patients with CEA ≤ 4 was significantly higher than CEA > 4 (49 vs 26 months, *p* value 0.041) and Median survival of patients with CA19–9 ≤ 37 was significantly higher than CA19–9 > 37 (49 vs 24 months, p value 0.019). (Table 4, Fig. 2a and b] Similarly, female patients (*p* = 0.045), patients with stage T1/T2 (*p* = 0.001), node negative patients (*p* = 0.001) and no perineural invasion (*p* = 0.010) showed significantly better survival. Patients with lymphovascular invasion (present/absent) showed similar survival. However on multivariate analysis, none of the factors was found to be statistically significant in affecting overall survival. (Table 5).

**Table 4:** Factors affecting overall survival in patients undergoing curative resection (*n* = 86)

Variables		n	Median survival (months)	3 yr survival	5 yr survival	***P*** value (Log rank)
**Age**	**≤50**	40	50	62.2%	46.6%	0.051
	**> 50**	46	28	46.3%	22.5%	
**Sex**	**Female**	67	50	61.5%	56.4%	**0.045**
	**Male**	19	26	34.3%	11.4%	
**CEA**	**≤4**	35	49	64.1%	42.8%	**0.041**
	**> 4**	33	26	41.8%	12.5%	
**CA19–9**	**≤37**	59	49	66.6%	40.0%	**0.019**
	**> 37**	27	24	29.6%	–	
**T stage 1/2**		52	50	74.5%	49.7%	**0.001**
**T stage 3/4**		34	16	18.0%	09%	
**Node negative**		47	55	22.3%	08%	**0.001**
**Node positive**		39	24	77.4%	57.5%	
**LVI**	**No**	43	28	50.0%	37.5%	0.633
	**Yes**	17	26	30.3%	30.3%	
**PNI**	**No**	42	39	57.7%	43.3%	**0.010**
	**Yes**	18	16	15.7%	15.7%

**Table 5:** Univariate and multivariate analysis of factors affecting overall survival in patients undergoing curative resection

variables		n	Univariate		Multivariate	
			***P*** value	HR (CI)	***P*** value	HR (CI)
**Age**	**< 50**	40	0.058	0.520 (0.264–1.022)		
	**> 50**	46				
**Sex**	**female**	67	0.052	0.511 (0.260–1.006)		
	**Male**	19				
**CEA**	**< 4**	35	**0.049**	0.473 (0.225–0.996)	0.720	0.831 (0.302–2.285)
	**> 4**	33				
**Ca19.9**	**< 37**	59	**0.024**	0.463 (0.237–0.903)	0.274	0.574 (0.212–1.552)
	**> 37**	27				
**T stage1/2**		53	**0.001**	0.184 (0.093–0.365)	0.368	0.598 (0.195–1.831)
**T stage3/4**		33				
**Node Negative**		47	**0.001**	0.264 (0.132–0.524)	0.692	0.781 (0.229–2.659)
**Node Positive**		39				
**LVI**	**No**	43	0.639	1.211 (0.544–2.698)		
	**Yes**	17				
**PNI**	**No**	42	**0.014**	2.567 (1.212–5.440)	0.824	1.147 (0.343–3.830)
	**Yes**	18				

Among the resected patients (*n* = 86), on follow-up, recurrence was noted in 38 patients (44%). Among these 23(60.5%) patients had systemic recurrence alone, 5(13.2%) had local recurrence alone whereas 10(26.3%) patients had both systemic and local recurrence. On univariate analysis of patients having recurrence, CEA > 4 (*p* value = 0.040), CA19–9 > 37 (*p* = 0.013),T stage (T3/ T4, p value 0.001), N stage (N+ p 0.001) and perineural invasion positivity (p 0.012) were found to be statistically significant in predicting recurrence. On multivariate analysis, only CA19–9 > 37 (*p* value 0.020, hazard ratio 5.803) was found to be statistically significant. (Table 6).

**Table 6:** Univariate and multivariate analysis for factors affecting recurrence

Variables		Recurrence(n)	Univariate		Multivariate	
			***P*** value	HR (CI)	***P*** value	HR (CI)
**Age**	**< 50**	13	0.054	0.420 (0.174–1.016)		
	**> 50**	25				
**Sex**	**female**	26	0.072	0.379 (0.132–1.089)		
	**Male**	12				
**CEA**	**< 4**	11	**0.040**	0.352 (0.130–0.954)	0.906	1.093 (0.253–4.725)
	**> 4**	19				
**Ca19.9**	**< 37**	21	**0.013**	0.293 (0.111–0.770)	**0.020**	5.803 (1.324–25.440)
	**> 37**	17				
**T stage1/2**		13	**0.001**	10.989 (3.861–31.275)	0.412	2.111 (0.354–12.582)
**T stage3/4**		25				
**Node Negative**		12	**0.001**	5.667 (2.222–14.451)	0.484	1.874 (0.322–10.904)
**Node Positive**		26				
**LVI**	**No**	9	0.541	0.704 (0.228–2.171)		
	**Yes**	19				
**PNI**	**No**	13	**0.012**	0.214 (0.064–0.716)	0.804	0.788 (0.120–5.164)
	**Yes**	15				

On subgroup analysis of tumour markers in 32 patients who were diagnosed gall bladder cancer incidentally was performed. In metastatic group (*n* = 9), the median Ca 19.9 and CEA levels were 37.5 ng/ml (IQR-14.8-299.8); 5.7 IU/ml (IQR: 3.8–40.10) respectively. In unresectable group (*n* = 1) the Ca 19.9 and CEA levels were 12.56 ng/ml and 6 IU/ml respectively. In resectable group (*n* = 22), the median Ca 19.9 and CEA levels were 24.7 ng/ml (IQR: 10.1–35.6) and 3 IU/ml (IQR: 2.4–5.0). The Ca 19.9 levels were significantly higher in metastatic group than resectable group (*p* = 0.039). However CEA levels were not statastically significant in metastatic group compared to resectable group (*p* = 0.07).

## Discussion

GBC is a biologically aggressive disease with poor prognosis and late presentation. Despite screening from the outpatient department 38% patients of GBC without SOJ were found to have metastatic disease at the time of surgery as shown in our previous study [4]. Therefore, it is important to diagnose the advanced nature of the disease preoperatively, to avoid the morbidity related to anesthesia and surgery in these patients.

### Predicting metastasis

CA19–9 and CEA are the most commonly used tumor markers in GBC [9, 12]. The role of these markers in predicting metastasis or unresectability needs attention and evaluation. Finding a serum levels of these markers will help to increase the accuracy of predicting the prognosis of these patients. In a study by Wang et al., value of CA19–9 (but not CEA) increased gradually with progression of clinical stages [9]. The current study demonstrated that CA19–9 value increases as the tumor burden increases (*p* < 0.001) but the same was not true for CEA levels (*p* = 0.20). Thus, CA19–9 may be useful in predicting the tumor burden in these patients. In our study the cutoff value of CA19–9 > 72 and CEA > 5 had high specificity but low sensitivity in detection of metastatic disease with a AUROC of 0.67 and 0.62 for CA19–9 and CEA respectively. Liu et al. [13] found cut-off levels CA19–9 of 98.9 IU/ml, an independent predictor of resectability in GBC patients who underwent attempted resection with a sensitivity of 76.3% and specificity of 70.8%. They showed CA19–9 levels may predict R0 resection. Shukla PJ et al. [14] in their analysis of 335 patients, which also included 80 patients with jaundice, found that when CA19–9 levels exceeded 90 IU/ml, 95% patients had unresectable disease. However, they did not calculate the cut-off levels in their study. Although high levels of CA19–9 (> 70–100 IU/ml) have been associated with a high probability of unresectable disease, but one should not deny these patients a chance of surgical resection based on the level of tumor markers alone as surgery is the mainstay for curative treatment in these patients. Literature says PET-CT changes the surgical management in 17–23% of patients with GBC (Petrowsky et al. 2006, Corvera 2008 et al) [ 15, 16]. Therefore we suggest the use of PET-CT in work up alogirthm of patients when theCA19–9 is> 72 and CEA > 5 to detect occult metastasis preoperatively, potentially avoiding surgical exploration, particularly when routine imaging does not show any sign of unresectability.

### Prognostic marker

Wen et al. [11] used a time dependent ROC curve and AUROC for prognostic accuracy of the tumor markers. They found AUROC at 5 years for CA19–9 was 0.77, CEA was 0.76 and for combined markers was 0.79 and concluded that the combination of an elevated CEA and CA19–9 was an independent predictor of poor prognosis in GBC patients undergoing resection.

We found a statistically significant difference in the survival of patients with CA19–9 > 37 vs ≤37 IU/ml and CEA > 4 vs ≤4 ng/ml in univariate analysis along with other tumour characteristics. However both these factors were not significant in determining overall survival on multivariate analysis. The overall 5-year survival of patients with CEA ≤4 and > 4 was 42.8 and 12.5% respectively. Similarly, patients with CA19–9 ≤ 37 had a 5-year survival of 40%, whereas none of the patients with CA19–9 > 37 survived for more than 5 years despite curative surgical resection. These observations suggest that preoperative CA19–9 and CEA levels have a utility in prognostication of patients undergoing curative surgical resection. Agarwal et al. [17] noted a 4-year survival of 78%in GBC patients undergoing extended cholecystectomy (*n* = 33),when CA19–9 levels were less than 20 IU/ml vs 33% when CA19–9 levels were more than 20 IU/ml. They noted that median survival was not reached when CA19–9 levels were less than 20 IU/ml vs 12 months when CA19–9 levels were more than 20 IU/ml [17].

### Predicting recurrence

The elevated preoperative level of CA19–9 has been associated with a high risk of recurrence after curative resection of biliary tract carcinoma in a few published studies including the current study. However, the optimal cut off value of CA19–9 remains controversial. Chung et al. found an optimal cutoff value of preoperative CA19–9 to be 55 IU/ml to predict recurrence in their study [18]. However, Chung et al. included a mix population of all biliary tract cancers in their study (only 20% of patients had GBC) and approximately 40% of patients among them had jaundice. Liu et al. [8] reported a mean value of > 250 IU/ml as cut-off for CA19–9 in predicting recurrence. The higher value reported by Liu et al. could be because they calculated the mean value and not used the ROC curve, or could also be attributed to the differences in study population. Chung et al. [18] found that the recurrence occurred in 61% patients with CA19–9 > 55 compared to25% whenCA19–9 was < 55 IU/ml. In our study, recurrence occurred in 63% patients with CA19–9 > 37 compared to 35% in patients with CA19–9 < 37 reaffirming that high CA 19–9 has significantly higher recurrence after curative surgery. The high preoperative CA19–9 level could also be considered as a criterion for considering patient for adjuvant chemotherapy after curative surgical resection.

In our present study we found CEA > 4, CA19–9 > 37, presence of T3/T4 tumors, node positive disease and presence of perineural invasion were predictors of recurrence. But on multivariate analysis, only CA19–9 > 37 was found to be statistically significant in predicting recurrence after surgery.

CA19–9 was found to be a better tumor marker than CEA in our study, as its median value increases with tumor burden. It has a higher area under ROC for prediction of metastatic disease and is a significant factor in predicting recurrence. Recent studies done to understand the molecular mechanisms of GBC show that the eukaryotic initiation factors (eIFs) monitor protein translational processes via PI3K/AKT/mTOR pathway play a pivotal role in cell growth, proliferation, apoptosis and malignant transformation. Overexpression of eIF6s and Insulin growth factor-2 mRNA binding protein, (IMP2) have been significantly correlated with advanced tumor and shorter survival of gallbladder cancer patients. Thus, besides the tumor marker CEA and CA 19–9, eIF6 and IMP2 may be used as a prognostic biomarker for overall survival in GBC patients and might be used as a potential therapeutic approach in future [19, 20]. Chronic inflammation is risk factor for gall bladder carcinoma. IL-6, a pleiotrophic cytokine is involved in such inflammation and if chronically stimulated will have detrimental effects. However the IL-6 R alpha (interleukin-6 receptor alpha) expression in GBc patients has good prognosis [21].

The strength of this study are that we included complete spectrum (both resectable and unresectable) of GBC patients to derive at the significance level of the tumour marker. We excluded patients of GBC with SOJ as jaundice by itself can erroneously lead to raised CA19–9, confounding the interpretation of data. Our study is limited by its retrospective analysis of data, which is potentially exposed to selection bias. Secondly, Lewis antigen (non secretory group for CA19–9) was not taken in to account and this could have decreased the accuracy of CA19–9 in predicting advanced disease. There was a limited data on postoperative levels of Ca 19.9 and CEA. Hence the difference between preoperative levels and post operative levels could not be calculated.

## Conclusions

Raised CA19–9 and CEA predict metastatic disease in patients with GBC without jaundice with a good specificity and may help in prognostication of the patient. CA19–9 is overall better than CEA in predicting tumor burden and recurrence. Patients with raised CA 19–9 levels may be considered for preoperative PET before exploration and for adjuvant chemotherapy after resection.

## Data Availability

The datasets used and/or analysed during the current study are available from the corresponding author on reasonable request.

## Abbreviations

CEA: Carcinoma Embryonic Antigen
CA19–9: Carbohydrate Antigen 19–9
GBC: Gall Bladder Cancer
SOJ: Surgical Obstructive Jaundice
PET: Positron Emission Tomography
ROC: Receiver operating characteristic
AUROC: Area under ROC curve

## References

[1] Hundal R, Shaffer EA (2014). Gallbladder cancer: epidemiology and outcome. Clin Epidemiol.

[2] Hawkins WG, DeMatteo RP, Jarnagin WR, Ben-Porat L, Blumgart LH, Fong Y (2004). Jaundice predicts advanced disease and early mortality in patients with gallbladder cancer. Ann Surg Oncol.

[3] Kim SJ, Lee JM, Lee JY (2008). Accuracy of preoperative T-staging of gallbladder carcinoma using MDCT. AJR Am J Roentgenol.

[4] Mishra PK, Saluja SS, Prithiviraj N. Etal. Predictors of curative resection and long term survival of gall bladder cancer- a retrospective analysis. Am J Surg. 2017;214(2):278–86. 10.1016/j.amjsurg.2017.02.006 28233537

[5] National Comprehensive Cancer Network. Hepatobiliary cancer( version 2.2017). 2017.

[6] Hatzaras I, Schmidt C, Muscarella P, Melvin WS, Ellison EC, Bloomston M (2010). Elevated CA 19-9 portends poor prognosis inpatients undergoing resection of biliary malignancies. HPB.

[7] Strom BL, Maislin G, West SL, Atkinson B, Herlyn M, Saul S (1990). Serum CEA and CA 19-9: potential future diagnostic or screening tests for gallbladder cancer?. Int J Cancer.

[8] Liu F, Hu HJ, Ma WJ, Yang Q, Wang JK, Li FY (2019). Prognostic significance ofneutrophil-lymphocyte ratio and carbohydrate antigen 19-9 in patients withgallbladder carcinoma. Medicine (Baltimore).

[9] Wang YF, Feng FL, Zhao XH (2014). Combined detection tumor markers for diagnosis and prognosis of gallbladder cancer. World J Gastroenterol.

[10] Wen Z (2017). Elevation of CA19–9 and CEA is associated with a poor prognosis in patients with resectable gallbladder carcinoma, HPB.

[11] Zhu AX, Pawlik TM, Kooby DA, Schefter TE, Vauthey J-N (2017). Gallbladder. AJCC Cancer staging manual.

[12] Yu T, Yu H, Cai X (2014). Preoperative prediction of survival in resectablegallbladder cancer by a combined utilization of CA 19-9 andcarcinoembryonic antigen et al. Chin Med J.

[13] Liu F, Wang JK, Ma WJ, Yang Q, Hu HJ, Li FY (2019). Clinical value of preoperativeCA19-9 levels in evaluating resectability of gallbladder carcinoma. ANZ J Surg.

[14] Shukla PJ, Neve R, Barreto SG, Hawaldar R, Nadkarni MS, Mohandas KM, Shrikhande SV (2008). A new scoring system for gallbladder cancer (aiding treatment algorithm): an analysis of 335 patients. Ann Surg Oncol.

[15] Corvera CU, Blumgart LH, Akhurst T, DeMatteo RP, D'Angelica M, Fong Y, Jarnagin WR (2008). 18F-fluorodeoxyglucose positron emission tomography influences management decisions in patients with biliary cancer. J Am Coll Surg.

[16] Petrowsky H, Wildbrett P, Husarik DB, Hany TF, Tam S, Jochum W, Clavien PA (2006). Impact of integrated positron emission tomography and computed tomography on staging and management of gallbladder cancer and cholangiocarcinoma. J Hepatol.

[17] Agrawal S, Lawrence A, Saxena R (2018). Does CA 19-9 Have Prognostic Relevance in Gallbladder Carcinoma (GBC)?. J Gastrointestinal Cancer.

[18] Chung MJ, Lee KJ, Bang S, Park SW, Kim KS, Lee WJ, Song SY, Chung JB, Park JY (2011). Preoperative serum CA 19-9 level as a predictive factor for recurrence after curative resection in biliary tract cancer. Ann Surg Oncol.

[19] Golob-Schwarzl N, Wodlej C, Kleinegger F (2019). Eukaryotic translation initiation factor 6 overexpression plays a major role in the translational control of gallbladder cancer. J Cancer Res Clin Oncol.

[20] Kessler SM, Lederer E, Laggai S (2017). IMP2/IGF2BP2 expression, but not IMP1 and IMP3, predicts poor outcome in patients and high tumor growth rate in xenograft models of gallbladder cancer. Oncotarget..

[21] Kleinegger F, Hofer E, Wodlej C (2019). Pharmacologic IL-6Rα inhibition in cholangiocarcinoma promotes cancer cell growth and survival. Biochim Biophys Acta Mol basis Dis.

